# Geographic location of health facility and immunization program performance in Hoima district, western Uganda: a health facility level assessment

**DOI:** 10.1186/s12889-020-09859-z

**Published:** 2020-11-23

**Authors:** Nicholas Kwikiriza Magambo, Francis Bajunirwe, Fred Bagenda

**Affiliations:** 1 Department of Community Health, Mbarara University of Science and Technology, PO Box 1410, Mbarara, Uganda; 2 Directorate of Health services, Hoima District, P.O.BOX 2, Hoima, Uganda

**Keywords:** Immunization, Performance, Vaccine, Coverage, Location, Associated factors, Rural Uganda

## Abstract

**Background:**

Globally, immunization coverage for childhood vaccines is below the immunization target of achieving at least 90% coverage with the pentavalent vaccine. In Uganda, a recent survey shows 80% of districts had poor immunization program performance. However, there is significant variation in performance within and between districts. We hypothesized that geographic location of a health facility may influence performance of its immunization programs. Therefore, the purpose of this study was to examine whether geographical location of a health facility within a district is associated with performance of the immunization program in Hoima district, western Uganda.

**Methods:**

We conducted a cross sectional study using a mixed methods approach. The main study unit was a health center and we also interviewed health workers in-charge of the facilities and reviewed their health facility records. We reviewed the Uganda Health Management Information System (HMIS) 105 reports of six months to obtain data on immunization program performance. Performance was categorized using World Health Organization’s Reach Every District (RED) criteria and classified as poor if a facility fell in category 3 or 4 and good if 1 or 2. We also conducted key informant interviews with immunization focal persons in the district. We examined the association between dependent and independent variables using Fisher’s exact test.

**Results:**

We collected data at 49 health facilities. Most of these facilities (55.1%) had poor immunization program performance. Proximal location to the central district headquarters was significantly associated with poor immunization program performance (*p* < 0.05). Attitudes of health workers in the more urban areas, differences in strategies for outreach site selection and community mobilization in the rural and urban areas were suggested as possible explanations.

**Conclusions:**

Proximal location to the urban setting near district headquarters was strongly associated with poor immunization program performance. To be able to reach larger numbers of children for vaccination, interventions to improve performance should target health facilities in urban settings.

**Supplementary Information:**

The online version contains supplementary material available at 10.1186/s12889-020-09859-z.

## Background

According to the World Health Organization (WHO), immunization coverage for childhood vaccines is still below the optimum target of achieving 90% or more for coverage with 3 doses of pentavalent vaccine [1, 2]. The WHO also approximates that two million children die every year due to vaccine preventable diseases and approximately 30% of children globally lack access to vaccines. Children in sub-Saharan Africa are ten times less likely to access vaccines than children in developed countries [3]. In Africa 81% of unvaccinated infants were found in ten countries Uganda inclusive [4]. In Uganda, a WHO report showed 90 out of 112 districts or 80% had poor immunization performance [5]. In only 20% of the districts, at least 80% of the targeted children received all the recommended doses of the lifesaving childhood vaccines.

Several studies have been conducted to examine the factors associated with immunization program performance (IPP) and the challenges of vaccination programs in resource limited settings [6–10]. The key factors identified include those at individual level such as access to information on maternal and child health, socio-demographic characteristics of parents or guardians and level of education. Religious beliefs, traditional remedies and mistrust of western medicine also influence program performance [11]. Health system factors such as funding constraints, human resource factors such as health worker shortages, training deficiencies, poor attitude of health workers and vaccination teams, inadequate infrastructure, supplies and equipment, and structural factors such as long distance from health facility are critical for successful programs [12–14].

Although several studies have shed light on the multiple factors that may influence immunization performance, there is limited data on how geographic distribution of health facilities may influence immunization performance. Data show that even within the same district where health facilities share common structural barriers, there is variation in performance [15, 16]. We hypothesized that geographic distribution of the facilities may influence their performance. The DPT3 coverage for the rural district of Hoima on the shores of Lake Albert in Uganda was recently reported to be 19%, way below the national level [17]. Therefore, the aim of this study was to examine whether geographic distribution of health facilities influences immunization program performance by health facilities in this rural district of western Uganda.

## Methods

### Study design and setting

We conducted a cross sectional study with both quantitative and qualitative methods of data collection. First, we collected the quantitative data, and then the qualitative data were collected to provide meaning and explanation to the quantitative findings. The study was conducted at both public and private health centers in Hoima district, a predominantly rural district in western Uganda, bordering with the Democratic Republic of Congo. The district has a population of 572, 896 inhabitants and 3.9% of the population is below one year. The district has 49 public and private health facilities that offer immunization services.

The data on immunization is captured in the health management information system (HMIS 105), a health unit out-patient monthly report. The report includes monthly attendance data for out-patient department (OPD) including OPD cases diagnosed and managed, maternal child health services offered, HIV/AIDS service data, laboratory data, stock outs of essential drugs and supplies and financial data. Health centers compile the monthly HMIS 105 reports that are then entered in a Ministry of Health online electronic database, the district health information system 2 (DHIS2).

In our study, the sampling unit was a health facility. We included all the 49 public and private health facilities on Hoima district HMIS reporting list that offered immunization services in the previous six months prior to the study. In Uganda, health center II are units at the parish level, health center III at sub-county and health center IV is at county level. The private health centers in our study were one HC IV, five HC III and three HC II. All these health centers included in this study are accredited to provide static and outreach immunization services. Private health centers are supervised by the district health office and health-sub district offices. Uganda National Expanded program for Immunization (UNEPI) also organizes and provides support supervision visits to both private and government health centers.

A team from the district and the health sub-district (HSD) or county-level health region conduct quarterly support supervision to the health centers. The Ministry of Health/UNEPI conduct quarterly support supervision to districts. Supervisors are expected to use supervision checklists developed by Ministry of Health. The tools assess for planning, implementation, cold chain maintenance, management of vaccines and EPI supplies and the program performance.

### Data collection

We collected data in three forms: 1) Immunization data at health centers from HMIS 105 reports 2) Health facility level assessment; (see Interview guide attached as supplementary material) and 3) Qualitative interviews with key informants. The immunization data at health centers are managed by records assistants and the in-charges of the health facilities. The records assistants hold a certificate in Records Management while the health center in-charges hold a diploma or degree in Clinical Medicine. We engaged the records assistants as research assistants to collect data from the health centers. The records assistants conducted the data abstraction of the immunization data. The research assistants were trained on the data collection tools and these tools were pretested at three health centers. We hired an independent research assistant to conduct the health facility level assessment. The key informant interviews were conducted by the lead investigator (NMK) and supported by one research assistant with a degree in social sciences.

### Measurements

Data to assess immunization program performance were obtained by reviewing HMIS 105 reports in the District Health Information System version 2 (DHIS2) that spanned a period of six months from January to June 2017 and we reviewed these reports to assess immunization program performance. The variables collected from HMIS reports included number of Expanded Program for Immunization (EPI) outreaches conducted, number of children who received the first and third dose of pentavalent vaccine and DPT3.

The DPT1 and DPT3 data were used to calculate immunization program performance according to the reach every district (RED) categorization criteria [18, 19]. The RED strategy was introduced by the WHO in 2002 with a goal of increasing immunization coverage to at least 90% with all vaccines in every district and country. It uses the pentavalent vaccine coverage and dropout rate to determine immunization program performance of health facilities. Specifically, DPT1 to DPT3 dropout rate was used to assess utilization of immunization services. The dropout rate was calculated as (cumulative DPT1- cumulative DPT3) *100/cumulative DPT1. The DPT1 coverage was calculated as (cumulative DPT1 * 100)/population of children under 1 year. The recommended DPT1 coverage is 90% and the drop-out rate is 10%.

Based on the recommended DPT coverage and drop-out, there are four categories of the RED classification namely; category 1 = high coverage (> 90% coverage) + low dropout rate (< 10%), category 2 = high coverage (> 90%) + high dropout rate (> 10%), Category 3 = low coverage (< 90%) + low dropout rate (< 10%) and Category 4 = low coverage (< 90%) + high dropout rate (> 10%). Health facilities in categories 3 and 4 are classified as having poor immunization performance, while categories 1 and 2 have good immunization performance.

We also conducted interviews with health workers in-charge of the health facilities in Hoima district using structured questionnaires to assess facility level factors that support immunization such as support supervision, organization of outreaches, financing, payment for outreaches, stock out of vaccines and cold chain maintenance. We collected data on demographic characteristics of health center in-charges like age, sex, marital status, years of experience, education level, and Expanded program for Immunization (EPI) training.

We also collected data on the characteristics of the health facility such as level of health center, ownership (government or private facility) and geographic location, presence of EPI outreach schedule, functional EPI fridge and its maintenance, means of transport, community mobilizer, payment of allowances for EPI activities, staffing level, conducting outreaches, discussing EPI performance in staff meetings, maintenance, stock out of vaccines and stock out of gas for EPI fridges. After preliminary analysis of quantitative data to assess immunization program performance at the health facilities, we conducted key informant interviews with relevant district and health sub-district officials knowledgeable about the vaccination program using open ended questionnaires.

### Data analysis

We checked the data for completeness, coded and entered them into Epi-Info version 7 and exported to Stata version 11 for analysis. The primary outcome for the analysis was immunization program performance as assessed based on the WHO’s RED category. The number of facilities was small and therefore to assess the association between dependent and independent variables, we used Fisher’s exact test. This test is robust for analysis involving small sample sizes where the expected number of observations in some of the cells in the contingency table is less than five [20]. Qualitative data were transcribed and no translations were necessary as data were collected in English. We manually read the transcripts and analyzed them using a thematic approach. We did not have any *apriori* themes. We also looked to the qualitative data to explain the gaps observed from the quantitative data.

### Ethical considerations

The study was approved by Mbarara University of Science and Technology (MUST) Faculty of Medicine Research Committee and MUST Research Ethics committee (MUST REC Protocol number 07/05–17). We obtained administrative clearance from Hoima district health office before initiation of the data collection process. Participants provided written informed consent before participating in the study.

## Results

### Characteristics of the health facilities

Forty-nine health centers were included in the study, 17 HC II (34.7%), 28 HCIII (57.1%), 3 HC IVs and 1 Regional referral hospital as shown in Table 1 below. Majority (40 or 81.6%) of health centers were government owned and the rest (18.4%) were private health facilities.

**Table 1:** Characteristics of health centers in Hoima District

Characteristic		Frequency (%)			*p* value
		Total	= < 25Km from District Head quarters	>25Km from District Head quarters	
Health Sub District	Bugahya	16 (32.7)	10 (37.0)	6 (27.3)	
	Buhaguzi	23 (46.9)	7 (26.0)	16 (72.7)	< 0.001
	Hoima Municipality	10 (20.4)	10 (37.0)	0 (0.0)	
Level of Health Centre	Regional Referral Health center IV Health center III Health center II	1 (2.1) 3 (6.1) 28 (57.1) 17 (34.7)	1 (3.7) 3 (11.1) 12 (44.4) 11 (40.7)	0 (0.0) 0(0.0) 16 (72.7) 6 (27.3)	0.11
Presence of EPI fridge	No Yes	5 (10.2) 44 (89.8)	3 (11.1) 24 (88.9)	2 (9.1) 20 (90.9)	0.99
EPI Fridge maintenance	No Yes	10 (20.4) 39 (79.6)	3 (11.1) 24 (88.9)	7 (31.8) 15 (68.2)	0.09
Stock out of Vaccines	No Yes	6 (12.2) 43 (87.8)	4 (14.8) 23 (85.2)	2 (9.1) 20 (90.9)	0.68
Stock out of Gas for EPI fridge	No Yes N/A^a^	21 (42.8) 9 (18.4) 19 (38.8)	14 (51.9) 4 (14.8) 9 (33.3)	7 (31.8) 5 (22.7) 10 (45.5)	0.48
Presence of EPI outreach schedule	No Yes	9 (18.4) 40 (81.6)	5 (18.5) 22 (81.5)	4 (18.2) 18 (81.8)	0.99
Received funding for EPI activities	No Yes	5 (10.2) 44 (89.8)	1 (3.7) 26 (97.3)	4 (18.2) 18 (81.8)	0.16
EPI allowances paid timely	No Yes	11 (22.4) 38 (77.6)	4 (14.8) 23 (85.2)	7 (31.8) 15 (68.2)	0.18
Presence of Community mobilizer	No Yes	1 (2.0) 48 (98.0)	1 (3.7) 26 (97.3)	0 (0.0) 22 (100)	1.0
Facility has means of transport	No Yes	34 (69.4) 15 (30.6)	22 (81.5) 5 (18.5)	12 (54.5) 10 (45.5)	0.06
EPI performance discussed in staff meetings	No Yes	10 (20.4) 39 (79.6)	7 (25.9) 20 (74.1)	3 (13.6) 19 (86.4)	0.48
Attend EPI performance review meeting	No Yes	25 (51.0) 24 (49.0)	13 (48.1) 24 (51.9)	12 (54.5) 10 (45.5)	0.18
Have support supervision	No Yes	5 (10.2) 44 (89.8)	1 (3.7) 26 (96.3)	4 (18.2) 18 (81.8)	0.16

^a^ Health centers do not have gas fridge

We found that 89.8% of the health centers had fridges for storage of vaccines. Most (87.8%) health centers reported to have experienced stock out of vaccines. All health centers had EPI focal persons. Immunization outreach schedules were present in 81.6% of health centers. Allowances for EPI had been paid in 77.6% of health centers. Support supervision had been conducted at least once in the previous six months in 89.8% of the health centers.

The characteristics of the health facilities are broken down by distance of less than 25 km or more and are shown in details in Table 1 below. Facilities in Buhaguzi were much further away from the headquarters compared to other health sub districts. Health facilities that were closer compared to those that were further away did not differ in the rest of the characteristics such as presence of an EPI fridge, vaccine stock-out, payment of allowances, and having support supervision visits.

### Demographic characteristics of respondents/health center in-charges

We interviewed one health worker per health facility included in the study and the demographics are shown in Table 2 below. Almost 50% of them were aged 30 years or less. Only 6 health workers with a degree headed a health facility and at least 60% of the health facilities were headed by diploma holders. Majority (*n* = 38 or 77.5%) were trained in EPI. Also majority of these health workers were male.

**Table 2:** Demographic characteristics of health facility in-charges

Characteristic		Frequency (%)
Age	≤30 years	23 (46.9)
	> 30 years	26 (54.1)
Years of experience	≤5 years	25 (51.0)
	> 5 years	24 (49.0)
Gender	Male	27 (55.1)
	Female	22 (44.9)
Title position	Medical doctor	3 (6.1)
	Clinical Officer	24 (48.9)
	Nursing Officer	7 (14.3)
	Enrolled Nurse or Midwife	13 (26.6)
	Others	2 (4.1)
Level of Education	Certificate	13 (26.5)
	Diploma	30 (61.2)
	Bachelors or Masters	6 (12.3)
Marital Status	Single	10 (20.4)
	Married	38 (77.5)
	Divorced	1 (2.1)
Trained in EPI	No	11 (22.5)
	Yes	38 (77.5)

### Performance and distribution of health facilities in Hoima district

**Fig. 1:**
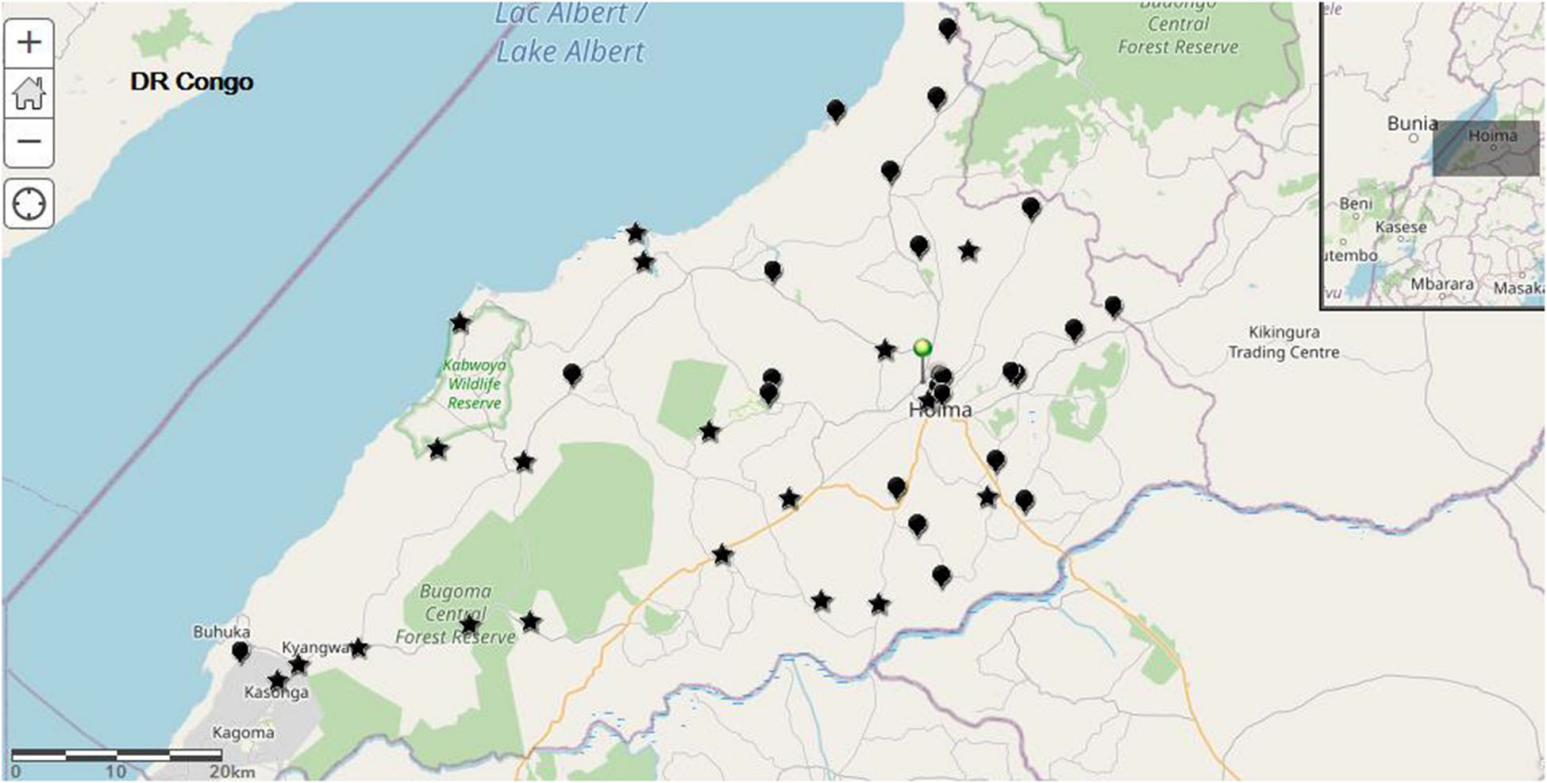
Map of Hoima district with health facilities assessed for immunization performance. The pin shows the urban center of the district headquarters, stars represent facilities with good performance and raindrops are facilities with poor performance. Source: Uganda Bureau of Statistics; https://ubos.maps.arcgis.com/home/index.html

Thirteen or 26.5% of the health facilities scored in the highest RED category and 11 scored in the lowest as shown in Table 3 below. At least 55% of the health facilities were rated as having poor immunization performance. The distribution of health facilities by their immunization performance is shown in Fig. 1 below in the map of Hoima. The map shows the clustering of health facilities with poor performance around or near the district headquarters, and those with good performance away from the headquarters.

**Table 3:** Performance of Health centers in Hoima district

Characteristic		Frequency (%)
**WHO RED Category**	**1**	13 (26.5)
	**2**	9 (18.4)
	**3**	16 (32.7)
	**4**	11 (22.4)
**Immunization performance**	**Good**	22 (44.9)
	**Poor**	27 (55.1)

### Bivariate analysis of factors associated with immunization performance

Several factors related to vaccine delivery were not significant and these include presence of an EPI fridge at the health facility, history of stock out of vaccines or gas for EPI fridge, presence of EPI outreach schedule, having received funding for EPI activities and paid allowances. Discussion of EPI performance at staff meetings, attendance of EPI performance review meetings, community mobilization and having means of transport for vaccination outreaches were not related to immunization program performance.

Having a community mobilizer, discussion of EPI performance in staff meetings, having at least 75% staffing level at the health facility and having conducted at least 20 outreaches in the last 6 months or conducting at least 75% of all planned outreaches for immunization were all unrelated to program performance.

There was no significant difference in the performance by the level of the health facility or ownership type and the results are shown in Table 4 below. However, facilities located in the Municipality or the more urban part of the district were more likely to perform poorly compared to those located distally such as Buhaguzi (*p* = 0.026). Although majority of the health facilities reported they received support supervision, we observed that facilities that received no support supervision were more likely to report good performance.

**Table 4:** Bivariate analysis of health center type and geographical location with immunization program performance

Characteristic		Total (***n*** = 49)	Good performance ***n*** = 22 (%)	Poor performance ***n*** = 27 (%)	*p* value^a^
Level of health facility	HC II HC III HC IV or higher	17 28 4	7 (41.2) 13 (46.4) 2 (50.0)	10 (58.8) 15 (53.6) 2 (50.0)	0.91
Ownership	Government Private	40 9	17 (42.5) 5 (55.6)	23 (57.5) 4 (44.4)	0.71
Support supervision	No Yes	5 44	5 (100.0) 17 (38.6)	0 (0.0) 27 (61.4)	0.009
Health Sub District	Bugahya Buhaguzi Municipality	16 23 10	4 (25.0) 15 (65.2) 3 (30.0)	12 (75.0) 8 (34.8) 7 (70.0)	0.026
Distance from District Headquarters	< 25KM >25KM	27 22	8 (29.6) 14 (63.6)	19 (70.4) 8 (36.4)	0.023

^a^Using Fishers exact test

The facilities that were located within 25 km of the district headquarters were more likely to perform poorly compared to those that were located much further away (*p* = 0.023 using Fisher’s exact test). At least 70% (*n* = 19) of the health facilities within 25 km performed poorly compared to only 36.4% (*n* = 8) of those more than 25 km from the district headquarters.

### Results of key informant interviews

We interviewed four key informants involved in immunization at the district and health sub-district. Overall three reasons were provided to explain the performance of the immunization program namely 1) health worker attitude 2) outreach site selection and 3) community mobilization as shown in Table 5 below.

**Table 5:** Reasons to explain immunization performance from key informant interviews

Reason	Explanation
Health workers’ attitude	Less time committed to immunization activities Residence near health facility
Outreach site selection	Convenient selection of outreach sites
Community mobilization	Outreaches conducted in the morning hours when clients are still in the gardens Less support for community mobilization Village health teams (VHTs) don’t reach all households with information on immunization dates Dates and time for outreaches not well known

### Health worker attitude

The key informants indicated that the health facilities located closer to the district headquarters might put up a poor performance because the health workers there reside in the town, away from their work stations and are more likely to commit less time to immunization activities like outreaches compared to their more rural counterparts. They mentioned that health workers in the rural areas reside close to the health facilities unlike their urban counterparts. The KIs mentioned that the urban health workers also tend to leave their work stations earlier to travel to town for other competing priorities as illustrated by one of the respondents:

“*Those health centres located next to Hoima town, many of their health workers reside in Hoima town and commute daily to work. Many of these leave their duty stations very early to return to their businesses in town*.” KI, Hoima district.

“*Health workers in urban areas go for immunization outreaches early morning and leave early when mothers are still in their gardens because they [health workers] want to return early to their families in town. But health workers in the rural areas commit more time from late morning [un]till evening for immunization outreaches*.” KI, Hoima district.

### Outreach site selection

The RED strategy requires that health centers conduct immunization outreaches to communities in hard-to-reach areas or villages located more than 5 km from the health center. KIs indicated that, health centers in the urban and peri-urban areas tended to select immunization outreach sites closer to the health center for their convenience.

“*Health workers in urban areas select outreach sites that they can easily access and many of them are located less than 5 km from their station, so they don’t delay to return*.” KI, Hoima.

### Community mobilization

All the key informants indicated that rural areas seem to have better mechanisms for community mobilization compared to their urban counterparts through grass root mechanisms.

“*The rural areas have implementing partners who support them in mobilizing communities using loud speakers and other local public address systems. In addition, these village women seem to have stronger interpersonal relationships and communication which they use to remind each other to attend immunization days*”. KI, Hoima district.

“*Health centres in rural areas rely on VHTs to mobilize communities for immunization. I have visited many households in urban and peri-urban areas and mothers reported no visits from VHTs. I have interacted with mothers in these areas and they usually know the location of the immunization outreach sites in their locations but usually don’t know the exact dates and time when health workers go there.*” KI, Hoima district.

## Discussion

In this cross sectional review of the immunization performance of health facilities in a district in western Uganda, over 50% of them were classified as having poor immunization performance. Proximal location of the health facility to the district headquarters and support supervision were associated with performance. Several factors related to the program such as infrastructure to deliver the vaccines and staffing levels were not significantly related to performance.

The poor performance in immunization at these health centers implies that many infants in this region in Hoima district and other districts with similar performance remain unvaccinated. The performance data support the findings of Uganda Demographic and Health survey (UDHS) of 2016 which showed that at least one in three children in Bunyoro region, where Hoima district is located, had not been received all basic vaccinations [21]. The same survey also found out that many regions in Uganda had significant proportion of infants who had not received all basic vaccinations. Our results are set in one district but provide insights into the performance on a much broader scale in the country. Several studies in sub Saharan Africa have reported less than the targeted levels for vaccination coverage [22–24].

Our data showed the health facilities that were located more remotely from the district headquarters or the more rural part of the district were likely to perform better than those located closer to the district headquarters. This inverse distance and performance relationship is striking and needs to be explained. Most health workers from health centers located close to the central district headquarters were reported to reside near the town center and were more likely to pursue competing economic activities in the municipality. Key informants suggested that this may cause them to commit less time to immunization activities and health facility related activities in general. Our key informant interviews suggested that the health workers at the remote health facilities conduct immunization outreach sessions at flexible hours for the rural residents who mostly spend their morning times in the gardens. The health workers in the urban areas do not provide this flexible option to the peri-urban dwelling clients, hence the shortfall. Our results agree with other studies elsewhere that show health worker attitudes and access are key influencers of immunization programs [25].

Another possible explanation for the poor performance in the urban setting is the less homogenous and more diverse urban populations, who are less stable and more mobile [26]. The Hoima district is undergoing rapid urbanization like most areas of Uganda, but might be more marked in this area due to recent oil explorations in the region and resultant in-migration of populations from neighboring districts seeking for opportunities. For instance, recent rural-urban migration has been associated with poor coverage [27].

Studies elsewhere have also shown rural-urban differences in performance of immunization programs [28]. Although urban areas are considered accessible and easy to reach, for immunization programs, populations in these areas should be considered among those with poor access to the services. For immunization to be successful, health workers need to maintain a certain level of contact with the recipient communities, [29] as a way to eliminate inequities.

The geographical differences in performance between the rural and urban counties may also be explained by the role of VHTs. Buhaguzi health sub district is more rural but hosts a refugee settlement which attracts some organizations that facilitate VHTs to mobilize communities for immunization, which services are absent in the urban counties. Also, the absence of health facilities in some urban parishes may play a role. For instance some divisions of Hoima municipality had many parishes that did not have a single health center. This implies that community members travel longer distances to access immunization sessions. Since most of immunization outreach sites were selected to the convenience of health workers, many children miss the opportunity to be vaccinated.

Our findings that health facilities in rural areas perform better than those in urban areas are supported by other studies elsewhere in sub Saharan Africa. For instance, a study done in the Gambia found that compared to the urban and peri-urban areas, children in the rural setting were more likely to be immunized based on the three antigens namely BCG, measles, and DPT and also as measured by full immunization by 12 months of age [30]. In the Gambia, poor performance in the urban areas was attributed to long queues at health facilities which served as a deterrent to attendance. In Indonesia, poor populations crowded in peri-urban clusters were less likely to be immunized, highlighting geographic distribution in immunization coverage [31]. Our study also shows the fewer health facilities in the urban parishes may be responsible for the poor performance. The fewer health facilities create inequalities in access to immunization services. Such inequalities were observed in a comparative study of immunization coverage in the urban slums of Ouagadougou and Nairobi and were responsible for the difference in vaccine coverage [32].

According to studies on immunization coverage in sub Saharan Africa, factors that affected immunization programs include support supervision, organization of outreaches, financing, logistics and cold chain maintenance, advocacy and communication, planning and management and use of surveillance data for decision making [33, 34]. However, our study did not find a significant association between immunization program performance and these factors.

Although support supervision has been associated with good immunization performance in studies from other sub Saharan African countries [33], our data show the opposite. We paradoxically found that facilities that did not have support supervision were all ranked as good performing. We consider this result spurious and to be taken with caution. The possible explanation for this unexpected finding is that majority of sites had regular support supervision, hence our data did not have sufficient variation to compare the sites with and without the supervision. To gain a better understanding of this finding, we triangulated these findings with qualitative data from key informant interviews and found out that support supervision was held irregularly. Also, we found that support supervision visits were integrated with other health programs and therefore never focused at details of immunization programs like review of performance data at the particular health facility. There is a potential bias in the assessment of the role of support supervision, if it was not being administered effectively. However, this finding could also have been due to bias arising from the possibility that support supervision might have targeted health centers with poor performance. Interventional studies have provided the evidence to show the benefit of support supervision in the delivery of health interventions [35].

The major strength of our study is we assessed health facilities in a remote district, and generated novel data for a district in an approach that is novel. Majority of studies have examined individual patient factors, but few have examined health facilities as a unit of observation. We have mapped the performance and showed the strong, albeit paradoxical association between proximity to the central district administration and immunization performance. Our study provided valuable data that will be used to inform immunization programs in many other districts of Uganda, and similar settings outside of Uganda. Our study has some potential weaknesses. First, this is not a national study, but a survey conducted in one district, hence the number of facilities assessed is small. For this reason, we were not able to conduct multivariable analyses as the models became unstable; nevertheless, the descriptive statistics and map data remain informative. However, given the similar rural-urban distribution of populations for majority of Ugandan districts, our results provide valuable lessons for other districts in the country and similar countries in sub Saharan Africa. Second, we used routinely collected data and these data have been shown to have weaknesses such as missing and incomplete entries [36] which may result in misclassification of the performance of the health facilities. The WHO commissioned a Strategic Advisory Group of Experts (SAGE) to examine the quality and use of global immunization and surveillance data. In their recent publication, the committee recommended investments in health systems factors such as personnel, processes, tools and governance in order to improve data quality [37]. Lastly, we did not assess for physical features such as mountains and roads, which are known to contribute to geographical access to services.

## Conclusions

In conclusion, our study has shown that majority of health facilities in this district of western Uganda had poor immunization performance. The health facilities located closer to the urban setting near the district headquarter s were associated with poorer immunization performance compared to those further away. Support supervision was negatively associated with immunization performance. However, the data are small and this finding should be interpreted with caution.

We recommend that future studies should be conducted in many districts and include a larger number of health facilities in order to provide a more comprehensive evaluation. Also, although we focused on health facility level assessment, more research is needed at individual level to explain performance. We also recommend monitoring and evaluation of immunization program performance at sub county and health sub district level to identify poor performing areas and put in place targeted interventions. There should also be dialogue meetings between community members and health workers as a way of involving community members in planning for immunization services like selection of site, time and date of outreach visits with much focus on areas with poor immunization performance.

The district health team needs to be mentored or trained and supported to conduct regular and planned support supervision of immunization programs in health centres, and not simply targeting places when performance has already gone down. Interventions are needed to improve involvement of VHTs in urban areas in mobilization for vaccination.

## Supplementary Information

**Additional file 1.** Interview Guide.

## Data Availability

All data supporting our findings are contained in the paper. There are some restrictions to data access and reasonable requests will be considered after approval from Mbarara University Research Ethics Committee. Data requests may be made to Nicholas Magambo Kwikiriza (corresponding author), Department of Community Health, Mbarara University of Science and Technology, PO Box 1410, Mbarara, Uganda, email: kwikiriza@gmail.com Tel: + 256783783288.

## Abbreviations

ANC: Antenatal Care
DHIS: District Health information system
DHO: District health officer
DHT: District Health team
DPT: Diphtheria Pertussis and Tetanus
EPI: Expanded Program on Immunization
HC: Health Center
HIS: Health Information System
HMIS: Health management information systems
HSD: Health sub district
ICT: Information communication technology
M&E: Monitoring and Evaluation
MOH: Ministry of Health
*p*-value: probability value
REC: Reach Every child
RED: Reach Every District
UNEPI: Uganda National Expanded Programme on Immunization
UNICEF: United Nation Children’s Fund
VHT: Village Health Team
WHO: World Health Organization
