# Supplementary material for: Geographic location of health facility and immunization program performance in Hoima district, western Uganda: a health facility level assessment
Source: BMC Public Health. 2020 Nov 23;20:1764. doi: 10.1186/s12889-020-09859-z (PMC7686762; doi:10.1186/s12889-020-09859-z)
Supplement: Supplementary file 1 — Additional file 1. Interview Guide. [file 12889_2020_9859_MOESM1_ESM.docx]

Questionnaire

Id. No …….. Date ……………………………..

**Heath facility details**

Health Centre Unique ID No: ………………………level …… Sub-county……………...

HSD …………………………………………… District …………………………

Ownership: Government NGO/PNFP PFP

What is the distance between your health centre and district headquarters?

Less than 25KM 25-50KM More than 50Km

Number of staffs Approved staffing norm

**Details of Health centre in-charge**

Position: Consultant MOSG SMO MO SCO CO

SNO NO EN/MW N/A Others (specify)

Level of education: Certificate Diploma Bachelors

Postgraduate diploma Masters

Sex: Male Female Age (years) ……… Years of experience ………

Marital status: Single Married Divorced Widowed

**Factors associated with HMIS reporting**

Is there a HMIS focal person? Yes No

If yes who? Records assistant Clinical staff Support staff

Has the HMIS focal person been trained in HMIS? Yes No

If yes, how? Pre-service training In-service training

Years of experience of HMIS focal person ……….

Have you had stock out of HMIS 033b and 105 report forms in the last 6months?

Yes No

Have there been any Change of HMIS reporting tools in the last 1year? Yes No

If yes did this negatively affect HMIS reporting? Yes No

What is the deadline for submitting the following HMIS reports?

HMIS 033b: Mid-day Monday Different answer

HMIS 105: 7^TH^ of next month Different answer

In the table below select **yes or no** on the following statement affecting HMIS reporting

| Factor | Yes | No |
| --- | --- | --- |
| Too many variables in HMIS reports affect completeness and time of submission of HMIS reports |  |  |
| Most health workers who participate in filling of HMIS reports are not trained in HMIS |  |  |
| There is poor telephone network at this Health Centre |  |  |
| Most staff have poor attitude towards HMIS reporting |  |  |
| Did the district or other institutions conduct supervision on HMIS reporting in the last 1 year |  |  |
| Do health workers receive training before implementation of new HMIS tools |  |  |
| Is there adequate funding to support HMIS reporting |  |  |
| Has there been any HMIS data review meeting held in last 1 year |  |  |
| There is poor road network between the health centre and the district headquarter |  |  |

In the table below select **yes or no** on the following statement affecting Immunization performance

| Factor | Yes | No |
| --- | --- | --- |
| Is there an EPI outreach schedule |  |  |
| Is there an EPI fridge |  |  |
| Did the cold chain technician ever touch the EPI fridge in Jan-June 2016 |  |  |
| Did you have stock outs of any vaccine in Jan-June 2016 |  |  |
| Did you have stock outs of gas in Jan-June 2016 (**only for gas fridges)** |  |  |
| Did you receive funding for EPI activities from PHC or any other source |  |  |
| Where all allowances for EPI outreaches in Jan-June 2016 paid in time |  |  |
| Do have community mobilisers (VHTs) for EPI outreaches |  |  |
| Did the HC have transport means for EPI outreaches in Jan-June 2016 |  |  |
| Is EPI performance discussed in staff meeting |  |  |
| Is there an EPI focal person |  |  |
| Was EPI performance discussed in staff meetings in Jan-June 2016 (ask for minutes) |  |  |
| Has the district or HSD ever organize an EPI performance review meeting in the last 1 year |  |  |
| Did you have any support supervision on EPI by the HSD, district or any other agency in the last 6months |  |  |

**Review of submitted HMIS reports**

Information to be got from submitted HMIS 033b & 105 reports and DHIS2 for January to June 2016

| How many EPI outreaches were planned for Jan-June 2016 |  |
| --- | --- |
| How many EPI outreaches were conducted for Jan-June 2016 |  |
| Percentage of conducted versus planned EPI outreaches |  |

EPI performance

| Month | DPT1 | DPT3 |
| --- | --- | --- |
| Jan 2016 |  |  |
| Feb 2016 |  |  |
| March 2016 |  |  |
| April 2016 |  |  |
| May 2016 |  |  |
| June 2016 |  |  |
| **TOTAL** |  |  |
| **DPT1-DPT3 dropout = (Total DPT1-Total DPT3)** |  | |
| **DPT1-DPT3 dropout rate = (Total DPT1-Total DPT3)/Total DPT1** |  | |

**HMIS reporting**

Use data for January to June 2016

| Indicator | Observation | Comment in case of a missing HMIS report |
| --- | --- | --- |
| Number of HMIS 033b reports submitted timely (by mid-day of Monday) |  |  |
| Number of HMIS 033b reports completely filled |  |  |
| Number of HMIS 105 reports submitted timely (by 7^th^ of Next month) |  |  |
| Number of submitted HMIS 105 reports with all data on immunization filled |  |  |
|  |  |  |

**Data accuracy**

| Month | DPT1 in submitted HMIS 105 report | DPT1 physical count from tally sheets | Indicate **yes or no** if figures in HMIS 105 report are matching with physical count from tally sheets | Comment if tally sheets are missing |
| --- | --- | --- | --- | --- |
| Jan 2016 |  |  |  |  |
| Feb 2016 |  |  |  |  |
| March 2016 |  |  |  |  |
| April 2016 |  |  |  |  |
| May 2016 |  |  |  |  |
| June 2016 |  |  |  |  |
| **TOTAL** |  |  |  |  |
